# Supplementary material for: Direct bacterial transcript visualization illuminates spatiotemporal environmental adaptation of Mycobacterium tuberculosis in the lung
Source: Sci Adv. 2026 Aug 12;12(33):eaeb8377. doi: 10.1126/sciadv.aeb8377 (PMC13464648; doi:10.1126/sciadv.aeb8377)
Supplement: Supplementary file 1 [file sciadv.aeb8377_sm.pdf]

Science Advances
AAAS

Supplementary Materials for

Direct bacterial transcript visualization illuminates spatiotemporal environmental adaptation of *Mycobacterium tuberculosis* in the lung

Anna-Lisa E. Lawrence and Shumin Tan

Corresponding author: Shumin Tan, shumin.tan@tufts.edu

Sci. Adv. **12**, eaeb8377 (2026)
DOI: 10.1126/sciadv.aeb8377

This PDF file includes:

Figs. S1 to S9

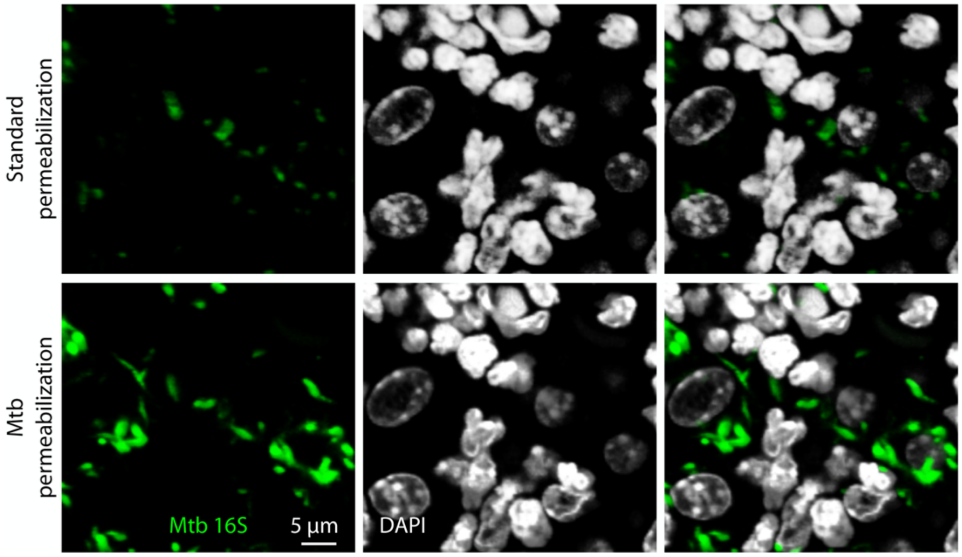

Fig. S1. Developed permeabilization protocol increases Mtb 16S rRNA signal brightness and ability to detect Mtb bacilli. C3HeB/FeJ mice were infected with Mtb for 2 weeks and lung tissues processed for HCR-FISH. 3D confocal images are shown, where the same section was first imaged after undergoing only a standard deparaffinization and antigen retrieval procedure, which includes a standard detergent treatment of 0.1% tween 20 for 4 minutes total after antigen retrieval, before probe hybridization ("standard permeabilization", top row). Subsequently, it was then processed using the developed permeabilization protocol for Mtb (see methods) and re-imaged ("Mtb permeabilization", bottom row). Nuclei (DAPI) are shown in grayscale and Mtb 16S rRNA in green.

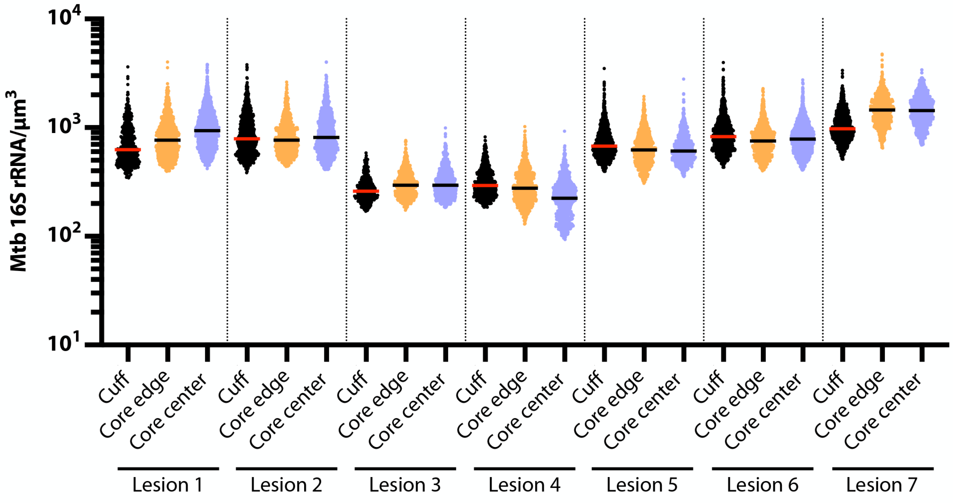

Fig. S2. Mtb in a subset of 16 wpi lesions exhibit lower 16S rRNA levels. C3HeB/FeJ mice were infected with Mtb for 16 weeks and lung tissues processed for HCR-FISH. Mtb 16S rRNA signal/µm^3 for individual bacteria or a group of tightly clustered bacteria, from 7 different lesions across 4 mice, were analyzed in each of the different lesion sublocations.

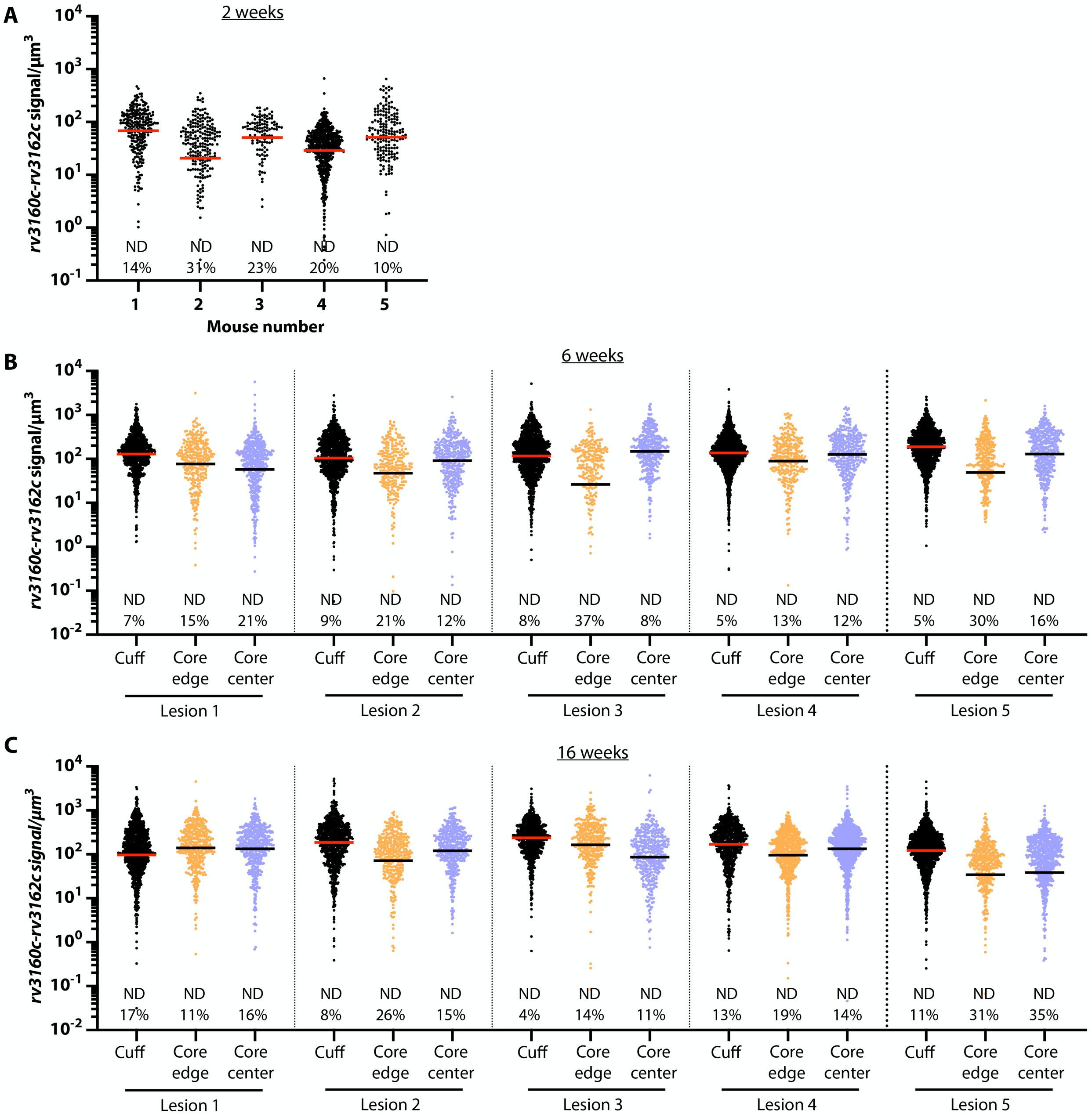

Fig. S3. Quantification of *rv3160c-rv3162c* expression in individual bacteria. *rv3160c-rv3162c* signal/µm^3 for individual bacteria or a group of tightly clustered bacteria at 2 (A), 6 (B), or 16 (C) wpi in C3HeB/FeJ mice. Horizontal lines mark the median for each sample, with the percentage of bacteria with non-detectable ("ND") levels of expression indicated at the bottom of each sample. For 6 and 16 wpi timepoints when necrotic lesions have formed, Mtb residing in the three lesion sublocations (cuff, core edge, or core center) were separately analyzed. Data are from 5 mice for 2 wpi, from 5 lesions from 5 mice for 6 wpi, and from 5 lesions from 4 mice for 16 wpi, obtained across 3 independent experiments.

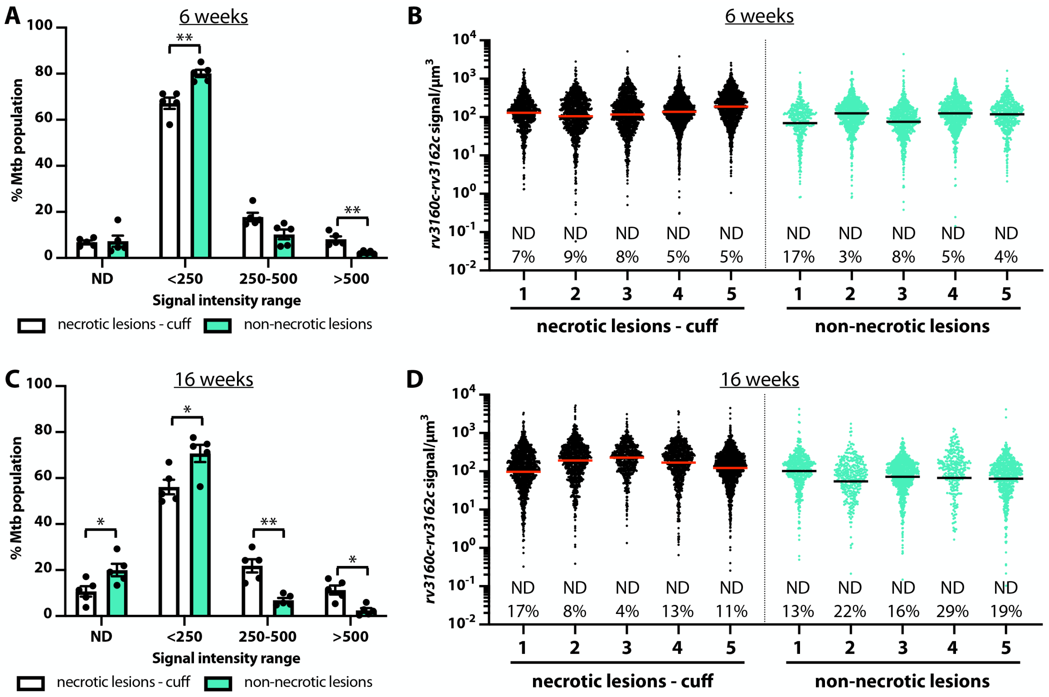

Fig. S4. Expression of Mtb lipid utilization genes is lower in Mtb residing in macrophages present in non-necrotic lesions versus in the cuff of necrotic lesions at 16 wpi. C3HeB/FeJ mice were infected for 6 (A and B) or 16 (C and D) weeks. The percentage of Mtb expressing different levels of *rv3160c-rv3162c* from non-necrotic lesions versus the cuff region of necrotic lesions at 6 (A) and 16 (C) wpi is shown, separated into 4 bins – non-detectable (ND), low (<250 signal/μm^3), intermediate (250-500 signal/μm^3), or high (>500 signal/μm^3). (B) and (D) show the quantification of *rv3160c-rv3162c* expression from individual bacteria at each indicated sublocation. Horizontal lines mark the median for each sample, with the percentage of bacteria with non-detectable ("ND") levels of expression indicated at the bottom of each sample. Data are from 5 lesions from 5 mice for 6 wpi, and from 5 lesions from 4 mice for 16 wpi, obtained across 3 independent experiments. Data from the cuff of necrotic lesions shown here are as shown in Fig. 3 and fig. S3, B and C. p-values were obtained with multiple unpaired t-tests with Holm-Sidak multiple comparisons test in (A) and (C). Only significant comparisons are indicated. * $p<0.05$, ** $p<0.01$.

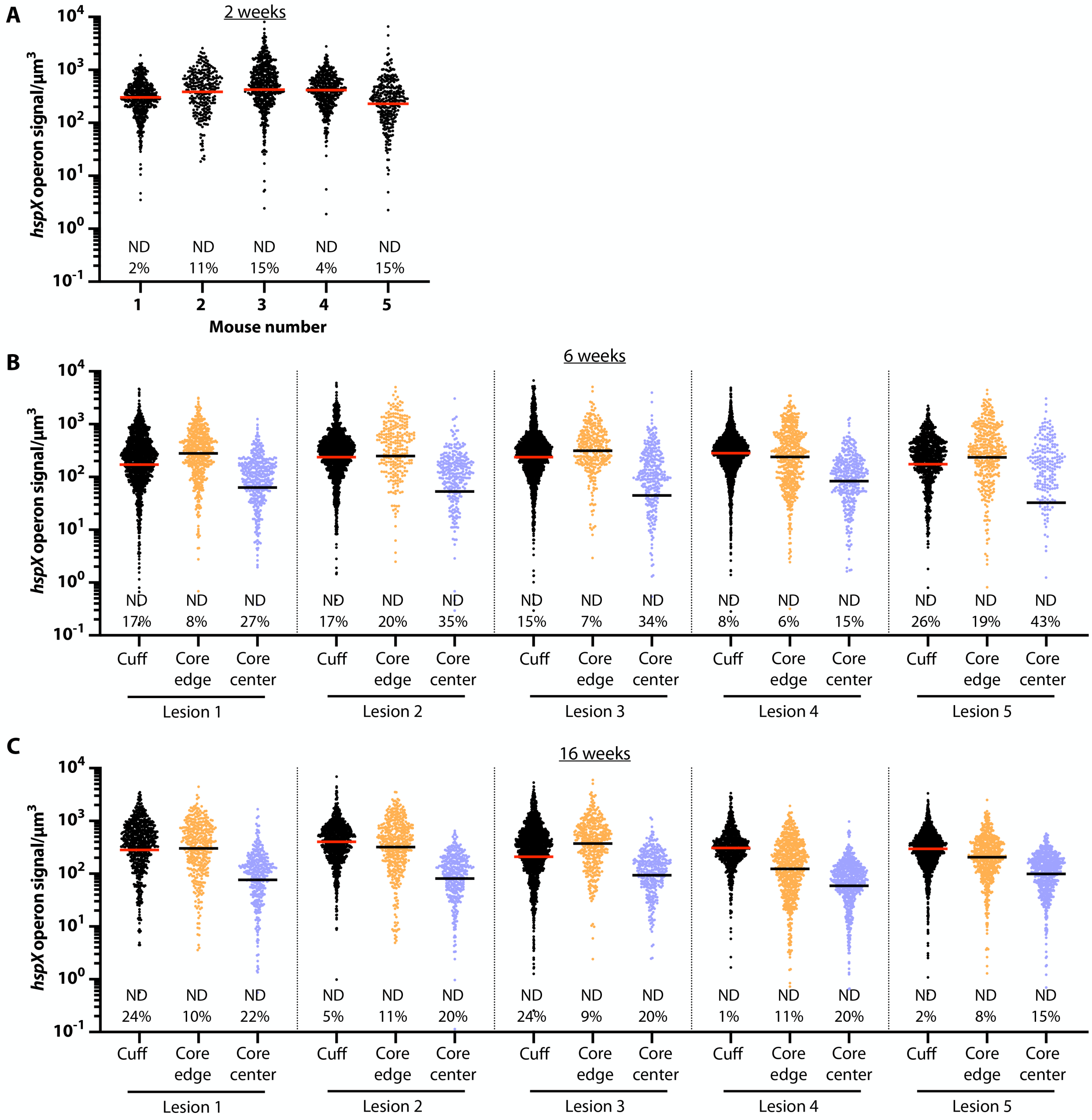

Fig. S5. Quantification of *hspX* operon expression in individual bacteria. *hspX* operon signal/µm^3 for individual bacteria or a group of tightly clustered bacteria at 2 (A), 6 (B), or 16 (C) wpi in C3HeB/FeJ mice. Horizontal lines mark the median for each sample, with the percentage of bacteria with non-detectable ("ND") levels of expression indicated at the bottom of each sample. For 6 and 16 wpi timepoints when necrotic lesions have formed, Mtb residing in the three lesion sublocations (cuff, core edge, or core center) were separately analyzed. Data are from 5 mice for 2 wpi, from 5 lesions from 5 mice for 6 wpi, and from 5 lesions from 4 mice for 16 wpi, obtained across 3 independent experiments.

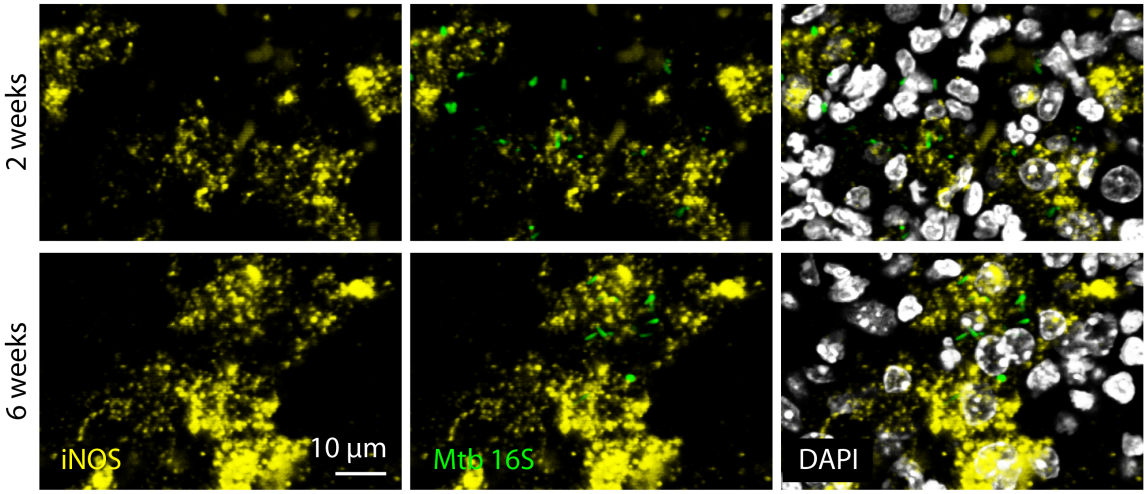

Fig. S6. ***Nos2*** **expression is observed in host cells even at 2 wpi.** 3D confocal images of 2 (top row) or 6 (bottom row) wpi lung Mtb-infected C3HeB/FeJ lung samples analyzed for *Nos2* expression (yellow) and Mtb 16S rRNA (green) via HCR-FISH. Nuclei (DAPI) are shown in grayscale.

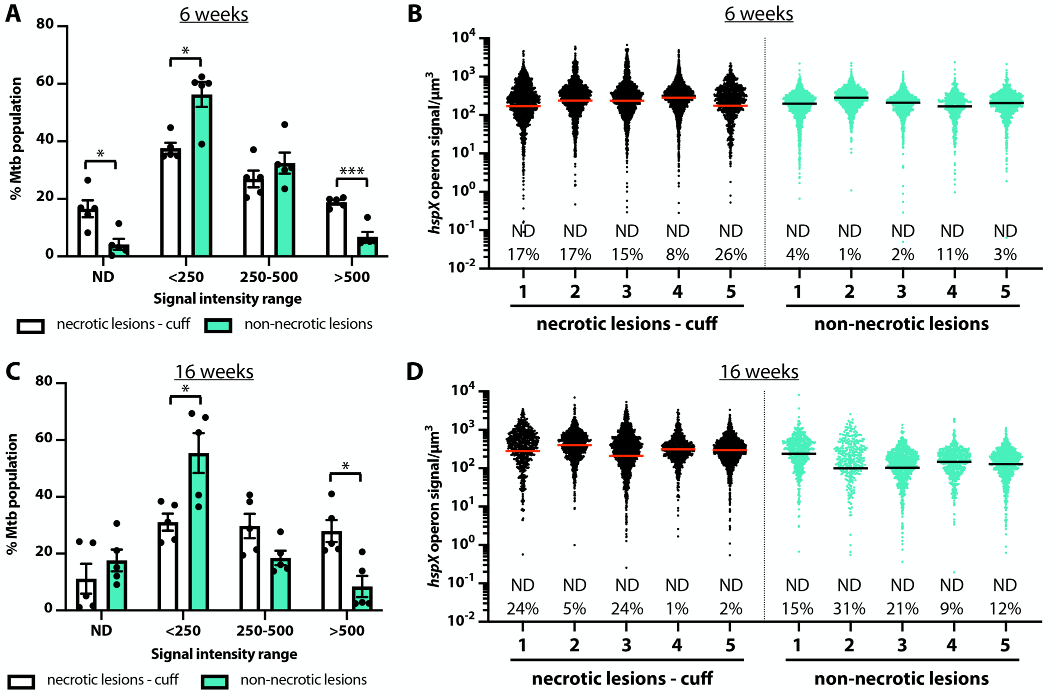

Fig. S7. Nitric oxide/hypoxia early responsive genes exhibit lower expression in Mtb residing in macrophages present in non-necrotic lesions versus in the cuff of necrotic lesions at 16 wpi. C3HeB/FeJ mice were infected for 6 (A and B) or 16 (C and D) weeks. The percentage of Mtb expressing different levels of the *hspX* operon from non-necrotic lesions versus the cuff region of necrotic lesions at 6 (A) and 16 (C) wpi is shown, separated into 4 bins – non-detectable (ND), low (<250 signal/µm^3), intermediate (250-500 signal/µm^3), or high (>500 signal/µm^3). (B) and (D) show the quantification of *hspX* operon expression from individual bacteria at each indicated sublocation. Horizontal lines mark the median for each sample, with the percentage of bacteria with non-detectable ("ND") levels of expression indicated at the bottom of each sample. Data are from 5 lesions from 5 mice for 6 wpi, and from 5 lesions from 4 mice for 16 wpi, obtained across 3 independent experiments. Data from the cuff of necrotic lesions shown here are as shown in Fig. 4 and fig. S5, B and C. p-values were obtained with multiple unpaired t-tests with Holm-Sidak multiple comparisons test in (A) and (C). Only significant comparisons are indicated. * $p<0.05$, *** $p<0.001$.

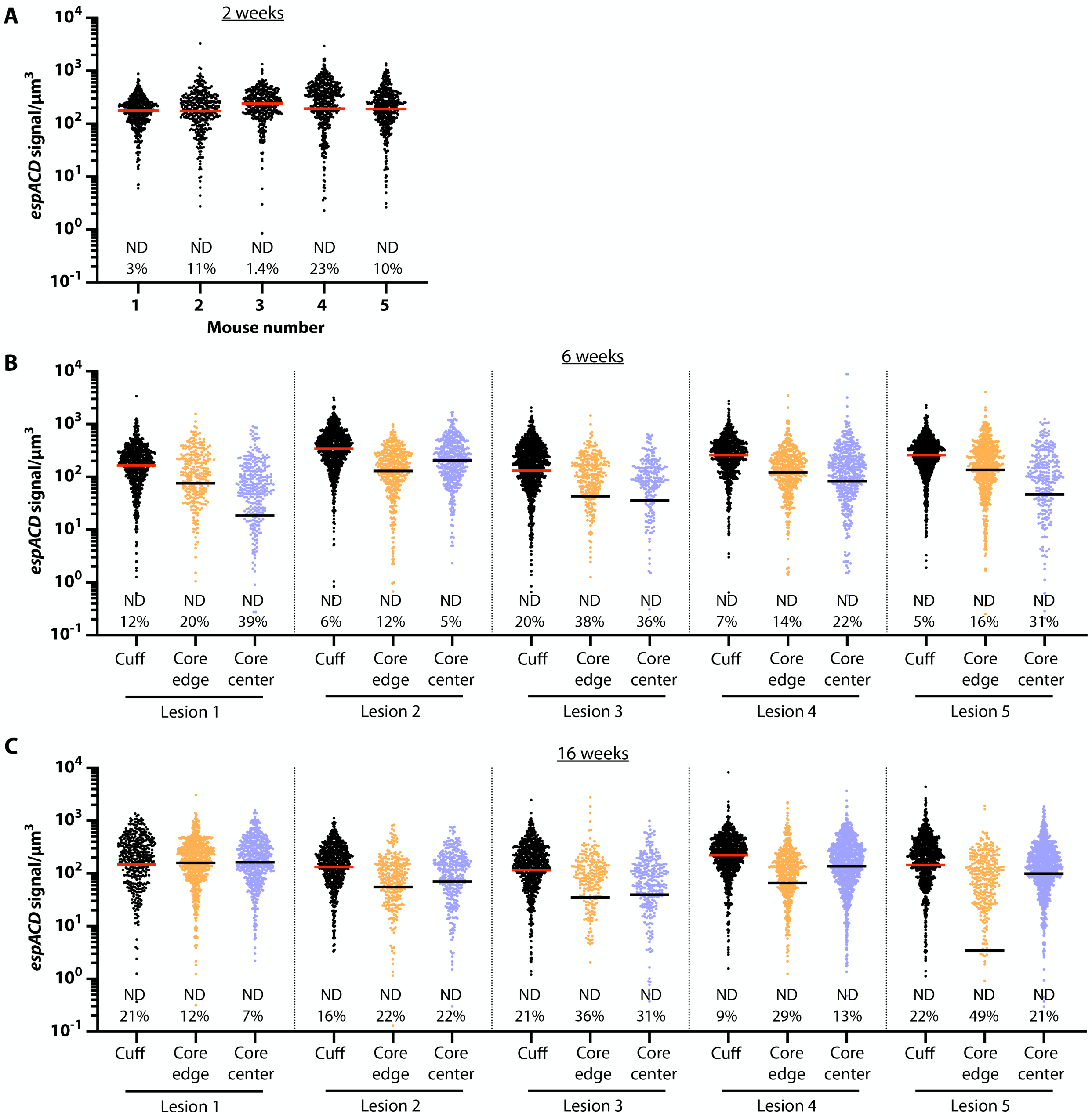

Fig. S8. Quantification of *espACD* operon expression in individual bacteria. *espACD* operon signal/µm^3 for individual bacteria or a group of tightly clustered bacteria at 2 (A), 6 (B), or 16 (C) wpi in C3HeB/FeJ mice. Horizontal lines mark the median for each sample, with the percentage of bacteria with non-detectable ("ND") levels of expression indicated at the bottom of each sample. For 6 and 16 wpi timepoints when necrotic lesions have formed, Mtb residing in the three lesion sublocations (cuff, core edge, or core center) were separately analyzed. Data are from 5 mice for 2 wpi, from 5 lesions from 5 mice for 6 wpi, and from 5 lesions from 4 mice for 16 wpi, obtained across 3 independent experiments.

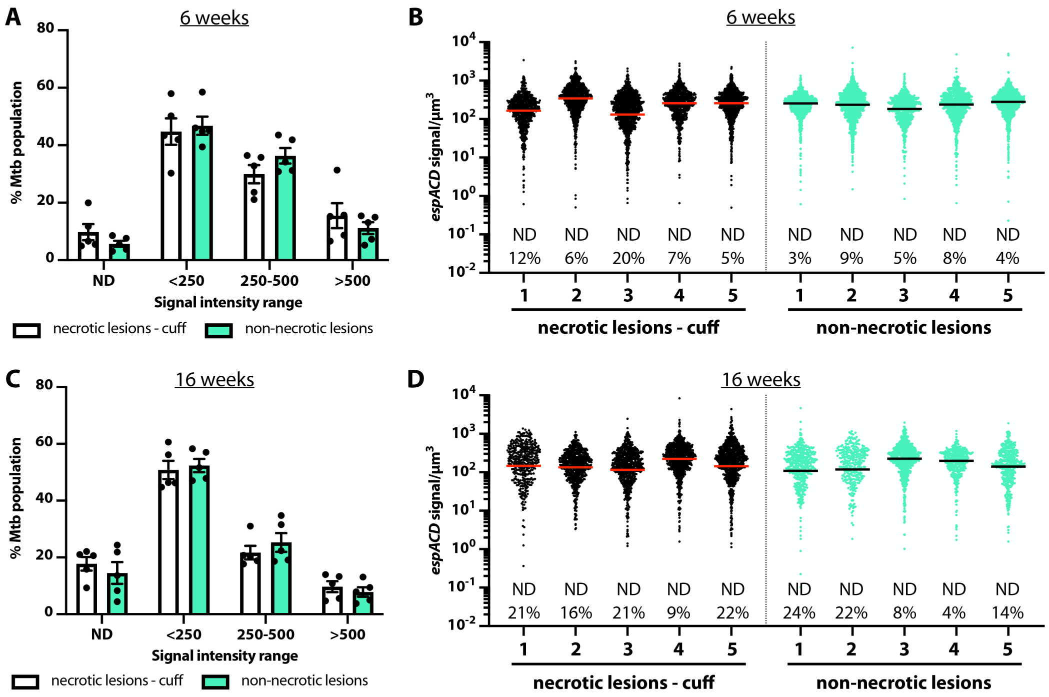

Fig. S9. ESX-1 type VII secretion system effector genes are expressed at similar levels in Mtb residing in macrophages present in non-necrotic lesions and in the cuff of necrotic lesions. C3HeB/FeJ mice were infected for 6 (A and B) or 16 (C and D) weeks. The percentage of Mtb expressing different levels of the *espACD* operon from non-necrotic lesions versus the cuff region of necrotic lesions at 6 (A) and 16 (C) wpi is shown, separated into 4 bins – non-detectable (ND), low (<250 signal/μm^3), intermediate (250-500 signal/μm^3), or high (>500 signal/μm^3). (B) and (D) show the quantification of *hspX* operon expression from individual bacteria at each indicated sublocation. Horizontal lines mark the median for each sample, with the percentage of bacteria with non-detectable ("ND") levels of expression indicated at the bottom of each sample. Data are from 5 lesions from 5 mice for 6 wpi, and from 5 lesions from 4 mice for 16 wpi, obtained across 3 independent experiments. Data from the cuff of necrotic lesions shown here are as shown in Fig. 5 and fig. S8, B and C. Multiple unpaired t-tests with Holm-Sidak multiple comparisons showed no comparisons were significant in (A) and (C).
